# Visfatin Promotes Renal Cell Carcinoma Progression: Evidence from Clinical Samples and Cell Line Models

**DOI:** 10.15586/jkc.v12i3.427

**Published:** 2025-09-26

**Authors:** Eiji Kashiwagi, Miho Ushijima, Shohei Ueda, Yoshihiro Sugita, Yui Mizushima, Takuo Matsukawa, Rieko Kimuro, Kazumasa Jojima, Katsuyoshi Higashijima, Yujiro Nagata, Akinori Minato, Ikko Tomisaki, Masatoshi Eto

**Affiliations:** 1 Department of Urology, Graduate School of Medical Science, Kyushu University, Fukuoka, Japan;; 2 Department of Urology, School of Medicine, University of Occupational and Environmental Health, Kitakyushu, Japan

**Keywords:** Adipose tissue, Adipocytokine, Perirenal fat, Renal cell carcinoma, Visfatin

## Abstract

The kidney is enveloped by perirenal fat, which secretes various hormones and cytokines, known as adipokines. Adipokines have been demonstrated to influence the development and progression of tumors, including renal cell carcinoma (RCC). Visfatin, an adipokine secreted by the adipose tissue, has been implicated in RCC, but its precise role remains unclear. In this study, we investigated the expression of visfatin in perirenal fat from patients with RCC and its correlation with the RCC malignant phenotype, and we examined the role of visfatin in RCC cell lines in vitro. This study included adipose tissue samples from 57 Japanese patients with clear cell RCC who underwent partial or radical nephrectomy. We examined the mRNA expression level of visfatin using real-time PCR. In vitro MTT assay and western blot were performed using human RCC cell lines. The mRNA expression of visfatin in peri-tumor versus peri-normal fat was higher in Fuhrman grade ≥2 cases compared with Fuhrman grade 1 cases. Furthermore, the addition of visfatin to RCC cell lines promoted cell proliferation, which was accompanied by increased protein expression of HIF1α, p-Akt, and p-ERK. Conversely, the addition of FK866, a visfatin inhibitor, suppressed cell proliferation and reduced these proteins. Our findings suggest that visfatin from peri-tumor adipose tissue influences the malignancy of RCC and plays a role in promoting the growth of RCC. This indicates a potential mechanism by which adipose tissue contributes to the progression of RCC, providing a possible target for therapeutic intervention.

## Introduction

Renal cell carcinoma (RCC) is one of the most common malignancies of the kidney and is known for its resistance to conventional chemotherapy and radiation therapy (1). The tumor microenvironment of RCC, including the surrounding adipose tissue, plays a significant role in tumor progression and metastasis (2). Adipose tissue is not only a storage site for fat but also an active endocrine organ that secretes various hormones and cytokines, collectively known as adipokines (3). The adipokine visfatin, also known as nicotinamide phosphoribosyltransferase (NAMPT), has garnered attention for its potential role in cancer biology (4).

Visfatin is highly expressed in visceral fat and has been implicated in various physiological and pathological processes, including inflammation, metabolism, and cancer (5, 6). Previous studies have suggested that visfatin may promote tumorigenesis by enhancing cell proliferation, inhibiting apoptosis, and modulating angiogenesis (6). However, the specific role of visfatin in RCC remains to be elucidated.

Given that adipokines like visfatin are secreted from perirenal fat, these molecules may influence not only tumor biology at the cellular level but also macroscopic tumor growth patterns. RCC grows in two distinct patterns: exophytic growth, where the tumor grows outward, and endophytic growth, where it grows inward into the kidney. These growth patterns may be influenced by various external signals and the tumor microenvironment. We previously reported that the morphology of perirenal fat affects the growth patterns of RCC (7). This suggests that exophytic and endophytic growth patterns may have different biological characteristics and associations with disease progression, potentially mediated by perirenal fat–derived factors.

In this study, we expanded our previous investigations and explored the potential role of visfatin in RCC. We investigated the expression of visfatin in peritumoral fat compared with normal perirenal fat in RCC patients and examined its effects on RCC cell lines.

## Materials and Methods

### Tissue collection and tissue processing

After receiving approval of the ethics (30-396) review board of Kyushu University Hospital, 57 patients with localized clear cell RCC (ccRCC) who underwent partial or radical nephrectomy were enrolled in this study. Patients with prior systemic therapy, metastatic disease at diagnosis, or insufficient tissue samples were excluded. We collected approximately 1 cm^3^ of peri-tumor and peri-normal adipose tissue from patients during partial or radical nephrectomy. Samples were immediately placed in 50 mL tubes without preservative solution and stored at −80°C within 1 h after collection.

### Real-time PCR (RT-PCR)

Total RNA was isolated from patient samples using ISOGEN (NIPPON GENE, Japan). First-strand cDNA was synthesized from 1.0 μg of total RNA using the Transcriptor First Strand cDNA Synthesis Kit (Roche Applied Science, Germany) in accordance with the manufacturer’s protocol. RT-PCR was performed using RT2 SYBR Green FAST Mastermix (Qiagen, Germany), as described previously (8). The primer sequences for RT-PCR were as follows: 5'-GGTGGAAAACACAGATCCAGA-3' (forward) and 5'-GTGGCCACTGTGATTGGATAC-3' (reverse) for visfatin mRNA; and 5'-GTGAAGGTCGGAGTCAACG-3' (forward) and 5'-GGTGAAGACGCCAGTGGACTC-3' (reverse) for GAPDH mRNA. Visfatin gene expression was normalized to GAPDH gene expression using the comparative 2-ΔΔCT method.

### Antibodies and chemicals

Anti-HIF1α (HPA001275), anti-p-Akt (ser473)(#4060), anti-Akt (#9272), anti-p-Erk (#4370), anti-Erk (#4695), and anti-TGFβ(#3711) antibodies were purchased from Sigma (St. Louis, MO, USA) and Cell Signaling (Danvers, MA, USA), respectively. Visfatin (SRP4908) and FK866 (F8557) were purchased from Sigma.

### Cell lines

Human RCC SN12C and KPK1 cells were established and cultured as described previously (9, 10). Human RCC ACHN cells were purchased from ATCC (Manassas, VA, USA) and cultured in Eagle’s minimal essential medium (Sigma). All culture media contained 10% fetal bovine serum. Cell lines were maintained in a 5% CO_2_ atmosphere at 37°C.

### MTT assay

We used the MTT (Thiazolyl Blue Tetrazolium Bromide) assay to assess cell viability, as described previously (11). Cells (2–6 × 10^3^) seeded in 96-well plates were cultured for 72 h in the presence of drugs and then incubated with 0.5 mg/mL of MTT (Sigma-Aldrich) in 100 µl of medium for 3 h at 37°C. MTT was dissolved by DMSO, and the absorbance was measured at a wavelength of 570 nm with background subtraction at 630 nm.

### Western blot

Equal amounts of proteins (30 µg) obtained from cell extracts were separated by sodium dodecyl sulfate-polyacrylamide gel electrophoresis and transferred to a polyvinylidene difluoride membrane. Western blot analysis was carried out using appropriately diluted antibodies, and the membrane was developed using a chemiluminescence protocol. Images were obtained using an image analyzer (LAS-3000 mini; Fujifilm, Tokyo, Japan).

### Statistical analysis

For statistical analysis, when multiple Fuhrman nuclear grades were present within a tumor, the predominant grade (i.e., the grade occupying the largest area) was used as the representative value. Log transformed fold changes of gene expression between groups were compared using the Welch *t*-test. Analyses were performed using R Statistical Software v4.3.1 (http://www.r-project.org/) and packages (12–15). P-values less than 0.05 indicated statistical significance.

## Results

### Visfatin mRNA expression is elevated in RCC tumors with a malignant phenotype

**Figure 1:**
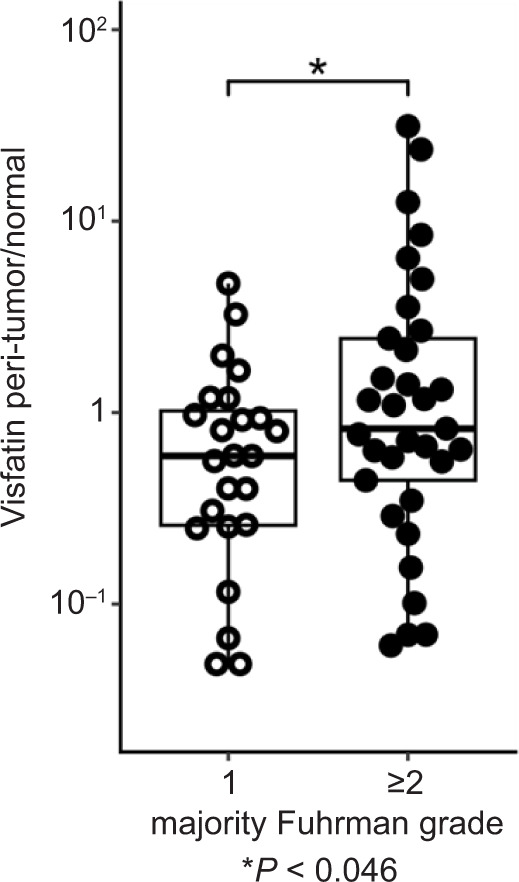
Ratio of visfatin mRNA expression in peritumoral to normal adipose tissue was compared between Fuhrman grade 1 and ≥2 RCC tumors. *P<0.05.

The characteristics of the 57 patients with RCC included in this study are summarized in Table 1. We evaluated visfatin mRNA expression by RT-PCR in adipose tissue adjacent to the tumor (tumor-associated adipose tissue) and compared it with expression in adipose tissue distant from the tumor (normal-associated adipose tissue). The ratio of these expression levels was then analyzed in relation to the nuclear grade (Fuhrman grade) of RCC. For statistical analysis, the predominant Fuhrman nuclear grade (the grade occupying the largest area) was used to represent each tumor.In cases with Fuhrman grade ≥2, the ratio of visfatin mRNA in peri-tumor fat versus peri-normal fat was higher in comparison with that in cases with Fuhrman grade 1 (Figure 1).

**Table 1:** Patient characteristics (n=57).

Variable	
Median age, years (range)	66 (39–82)
Gender, n (%)	
Male	41 (71.9)
Female	16 (28.0)
Laterality, n (%)	
Right	26 (45.6)
Left	31 (54.3)
Expansion pattern*, n(%)	
Outer	36 (63.1)
Inner	21 (36.8)
Hemodialysis, n (%)	1 (1.7)
pT stage, n (%)	
pT1a/1b	33(57.8)/16(28.0)
pT2a	2 (3.5)
pT3a/3c	5 (8.7) /1 (1.7)
Majority Fuhrman nuclear grade, n (%)	
1	24 (42.1)
2	32 (56.1)
3	1 (1.7)

* When a tumor was 50% or more exophytic, it was classified as an “outer location”, and if it was less than 50% exophytic, it was classified as an “inner location.”

### Visfatin promotes RCC cell proliferation, whereas the Visfatin inhibitor FK866 suppresses proliferation

**Figure 2:**
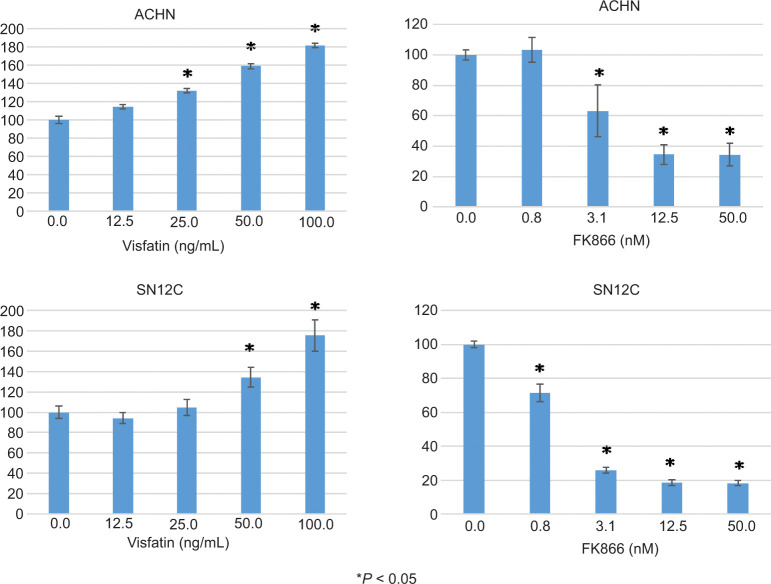
Visfatin promotes RCC cell growth. (A) ACHN and SN12C cells were cultured with the indicated concentrations of visfatin. After 72 h, the cell numbers were counted. The relative number of untreated cells was set as 100. All values are representative of at least three independent experiments. Data are shown as mean±S.D. *P<0.05 compared with untreated cells.

Next, we investigated the impact of visfatin on the cell proliferation of RCC cell lines. Visfatin induced cell growth in ACHN and SN12C RCC cell lines in a dose-dependent manner (Figure 2). By contrast, treatment with FK866, a visfatin inhibitor, inhibited cell proliferation.

### Visfatin upregulated the expression of proteins related to RCC growth while FK866 attenuated these effects

**Figure 3:**
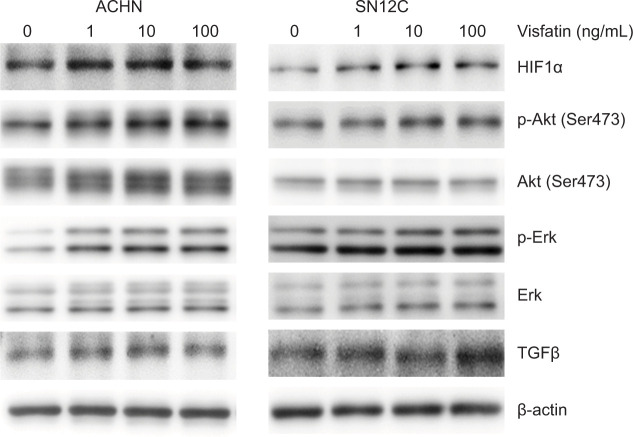
Visfatin upregulates HIF1α, p-Akt, p-Erk, and TGFβ expression. ACHN and SN12C cells were cultured with visfatin for 48 h, and HIF1α, p-Akt(Ser473), Akt, p-Erk, Erk, and TGFβ expressions were analyzed by western blotting. β-actin was used as a loading control.

**Figure 4:**
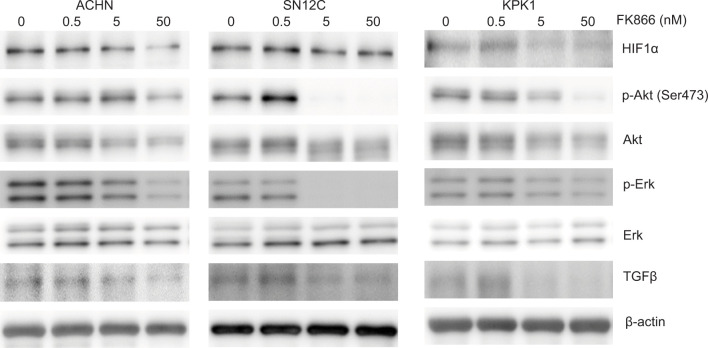
FK866 downregulates HIF1α, p-Akt, Akt, p-ERK, and TGFβ expressions. ACHN, SN12C, and KPK1 cells were cultured with FK866 for 48 h, and cell lysates were analyzed by western blotting with specific antibodies. β-actin was used as a loading control.

We then evaluated the protein expression of HIF1α, Akt, Erk, and TGFβ, which are known to be related to RCC growth (16–22) in RCC cell lines treated with visfatin or FK866. The results revealed that visfatin induced HIF1α, phospho-Akt (ser473), Akt, phospho-ERK, and TGFβ expression in ACHN and SN12C cells in a dose-dependent manner (Figure 3). FK866 attenuated those expressions (Figure 4).

## Discussion

In this study, we demonstrated that visfatin, an adipocytokine secreted from peri-tumor fat, may play a role in the malignant progression of RCC. Our data revealed that visfatin mRNA expression in peritumoral fat was significantly elevated in high-grade RCC compared with low-grade cases. Moreover, exogenous visfatin promoted cell proliferation in RCC cell lines, while its pharmacological inhibition by FK866 markedly suppressed tumor cell growth and signaling pathway activation.

Visfatin, also known as NAMPT, is a key regulator of nicotinamide adenine dinucleotide (NAD) metabolism that is involved in the pathophysiology of metabolic disorders and cancer cell metabolism (23). In this study, we also demonstrated that visfatin plays an important role in the metabolic regulation of RCC cells. The increased expression of HIF1α, p-Akt, and p-ERK in response to visfatin treatment indicates that visfatin activates key signaling pathways involved in cell proliferation, survival, and metabolism. HIF1α is a well-known regulator of the hypoxic response and has been implicated in promoting angiogenesis and metabolic adaptation in tumors (16, 17). The Akt and ERK pathways are critical for cell growth and survival (18, 19). TGFβ contributes to epithelial–mesenchymal transition, tumor-associated fibrosis, and immune evasion (20–22). Their induction or activation by visfatin suggests a potential mechanism by which visfatin promotes RCC progression.

Visfatin mRNA expression was shown to be higher in ccRCC samples than in adjacent normal tissue (24). In addition, visfatin is expressed at higher levels in plasma samples from patients with RCC compared with corresponding healthy controls, and there was a positive correlation between visfatin plasma level and T stage (25). The high expression of visfatin in ccRCC was also related to higher Fuhrman grade (26). These data suggest that visfatin is significantly correlated with the development and prognosis of RCC. Our current study demonstrates a correlation between elevated visfatin expression in peritumoral adipose tissue and higher tumor grade, without directly establishing a causal relationship. Whether elevated visfatin drives tumor progression or is a result of tumor-induced metabolic remodeling of surrounding fat remains to be clarified. This elevated expression may serve as a surrogate marker of tumor aggressiveness and suggests a potential role for visfatin or its downstream pathways as therapeutic targets. Temporal and mechanistic investigations using model systems to dissect this relationship are warranted. Nonetheless, the elevated expression of visfatin in peritumoral adipose tissue, together with its tumor-promoting effects demonstrated in vitro, suggests that visfatin may serve not only as a prognostic biomarker associated with tumor aggressiveness but also as a potential therapeutic target. Its inhibition suppressed key oncogenic pathways, supporting its role as a functionally relevant mediator of RCC progression.

Emerging data have indicated that ccRCC tumors actively engage with the adjacent perirenal fat, shaping a microenvironment that facilitates tumor progression. Wei et al. used both human and mouse models to show that ccRCC-derived parathyroid hormone-related protein (PTHrP) induces browning-like changes in neighboring perinephric adipose tissue by activating the protein kinase A (PKA) signaling pathway (27). This metabolic reprogramming of adipose tissue enhances thermogenic activity, which may in turn support tumor growth. These findings indicate that the interaction between ccRCC and perirenal fat is bidirectional. Not only does perirenal fat contribute to tumor progression through its secretion of adipokines and inflammatory cytokines, but ccRCC itself actively modulates the phenotype and function of the surrounding adipose tissue. Further temporal and mechanistic studies are needed to establish causality.

Several studies have reported elevated visfatin levels in various malignancies such as breast and colorectal cancers and the association with poor prognosis (28–30). Our findings add to the growing body of evidence, implicating a role for visfatin from peri-tumor fat in cancer pathophysiology for the first time and demonstrating its relevance in the tumor microenvironment of RCC. Our study focused on local visfatin expression in peritumoral fat, while previous reports have demonstrated elevated circulating visfatin levels in RCC patients and correlations with the tumor stage. This suggests that visfatin functions not only as a local paracrine factor but also as a systemic adipokine that influences tumor behavior. The relative contributions of local versus systemic visfatin should be explored in future research. Moreover, the suppression of these signals by FK866 suggests that visfatin’s enzymatic activity is critical for sustaining these malignant traits, and its inhibition may disrupt key tumor-promoting signaling networks.

The tumor microenvironment, particularly adipose tissue adjacent to tumors, has a critical role in cancer biology. The anatomical proximity of perirenal fat to renal parenchyma makes it a likely candidate for paracrine and metabolic crosstalk. Recent studies have reported that perirenal adipose tissue is not a passive depot but can actively secrete inflammatory cytokines and adipokines that modulate tumor behavior (31, 32). Perineal fat thickness and stranding have been reported to influence the progression-free survival of patients after surgical treatment of localized renal cancer (33). Perineal fat thickness was associated with the prognosis of patients with metastatic RCC receiving anti-VEGF therapy (34). Moreover, obesity and visceral adiposity have been independently linked to adverse outcomes in RCC, suggesting a systemic and local metabolic influence on tumor aggressiveness (35, 36).

FK866, a specific noncompetitive inhibitor of visfatin, has demonstrated anti-tumor efficacy in preclinical models by depleting intracellular NAD levels, leading to cell cycle arrest and apoptosis (37). Our findings support this mechanism in RCC and suggest that FK866 or related agents could be repurposed or developed as targeted therapies for RCC with elevated expression of visfatin, which has been documented in previous clinical studies (24, 25). A first-in-human phase 1 study of KPT-9274, a first-in-class dual inhibitor of PAK4 and NAMPT, in patients with advanced solid malignancies or non-Hodgkin’s lymphoma is ongoing (38). In spite of its promising anti-tumor effects, FK866 has shown dose-limiting toxicities in clinical trials because of systemic NAD+ depletion and bone marrow suppression (37, 39). These findings are consistent with our prior review, which highlighted the pro-inflammatory and tumor-promoting roles of perirenal fat in RCC and other systemic diseases (40). We proposed that perirenal fat is not only a passive structure but also an active participant in disease pathogenesis through the secretion of adipocytokines and cytokines, including visfatin, leptin, and TGF-β.

## Conclusion

Our study suggests that visfatin, derived from perirenal fat, may contribute to RCC progression by activating multiple pro-tumorigenic pathways. The inhibition of visfatin using FK866 attenuated cell growth and related signaling highlights its potential as a novel therapeutic target. Further in vivo studies and clinical validation are warranted to determine whether visfatin may serve as a predictive biomarker and therapeutic target in RCC. Our study findings are primarily derived from in vitro models and correlative analysis of human tissues. Further validation using in vivo models and clinical data is necessary. In particular, whether visfatin can serve as a predictive biomarker or therapeutic target with clinical significance remains to be determined.

In addition, we did not analyze patient characteristics such as BMI, metabolic status, comorbidities, or systemic inflammation, which may influence the expression of visfatin. These data were not consistently available in our cohort, and it indicates a limitation of the present study. Moreover, we did not evaluate patient survival, recurrence, or treatment response in relation to the expression of visfatin, and thus the prognostic value of visfatin could not be determined. Another limitation is that circulating (systemic) visfatin levels were not measured, so the relative contribution of local versus systemic sources remains unclear. Furthermore, other adipose-derived factors such as leptin, adiponectin, and resistin were not investigated in this study, and therefore the effects of visfatin may not be fully isolated.

Future studies should aim to distinguish between the local and systemic roles of visfatin and elucidate the bidirectional crosstalk between tumors and perirenal adipose tissue. Such investigations will advance our understanding of RCC pathogenesis and may identify novel targets for intervention.
